# AGXT-Driven Bile Acid Dysregulation Triggers Viral Gout in Astrovirus-Infected Jiangnan White Geese

**DOI:** 10.3390/vetsci12100951

**Published:** 2025-10-01

**Authors:** Suyu Fan, Xuming Hu, Wenxian Chai, Xiaoyu Shan, Yingjie Gu, Huangjun Shen, Guangzhong Peng, Wenming Zhao, Guohong Chen, Qi Xu

**Affiliations:** 1 Jiangsu Key Laboratory for Animal Genetic, Breeding and Molecular Design, College of Animal Science and Technology, Yangzhou University, Yangzhou 225009, China; fansuyu20030729@163.com (S.F.); lsshanxiaoyu218@163.com (X.S.); 19905184823@163.com (Y.G.); 13142662796@163.com (H.S.); 18797471288@163.com (G.P.); wmzhao@yzu.edu.cn (W.Z.); ghchen@yzu.edu.cn (G.C.); 2 Institute of Epigenetics and Epigenomics, College of Animal Science and Technology, Yangzhou University, Yangzhou 225009, China; 3 Changzhou Animal Disease Prevention and Control Center, Changzhou 213000, China; cwxian2025@163.com

**Keywords:** GAstV, bile acid metabolic, AGXT, goose

## Abstract

**Simple Summary:**

Goose astrovirus (GAstV) causes fatal gout in goslings, yet the underlying mechanism was unknown. We found that GAstV infection causes multi-organ damage, primarily targeting the kidney. Transcriptomic analysis revealed metabolic dysregulation, specifically an aberrant activation of bile acid metabolism. A key gene within this pathway, AGXT, was significantly upregulated. Since the AGXT enzyme influences uric acid production, its virus-induced overexpression is a likely driver of hyperuricemia and gout. This work unveils a novel metabolic mechanism for GAstV-induced gout, nominating AGXT as a promising target for breeding resistant poultry.

**Abstract:**

Goose astrovirus (GAstV) infection has emerged as a prevalent cause of urate deposition and viral gout in major goose farming across China, leading to high mortality and substantial economic losses. However, the molecular mechanisms linking GAstV to gout pathogenesis remain elusive. Here, a total of 10 five-day-old Jiangnan white goslings were selected, and tissue damage and kidney gene expression profiles were investigated. The results showed multi-organ damage in GAstV-infected gosling, including kidney, liver, spleen, and lung. Also, 342 differentially expressed genes were identified in infected kidney tissues after 10 days post-infection using transcriptomic sequencing, including 185 upregulated and 157 downregulated genes. In addition, gene set enrichment analysis revealed significant positive correlations between GAstV infection and bile acid metabolism and fatty acid metabolism pathways. Notably, bile acid metabolism was implicated in uric acid regulation and gout progression. Protein–protein interaction network analysis identified AGXT as a central hub gene within the bile acid metabolic pathway, with key upregulated interactors including PIPOX, ALDH1A1, and CAT. AGXT, a critical enzyme in glyoxylate detoxification, directly modulates uric acid biosynthesis. Our findings propose that GAstV-induced activation of bile acid metabolism, particularly AGXT upregulation, drives hyperuricemia and subsequent gout pathology. This study elucidates a novel mechanism of GAstV-associated metabolic dysregulation and provides actionable genetic targets for antiviral breeding strategies in waterfowl.

## 1. Introduction

Goose astrovirus (GAstV) infections has recently precipitated widespread outbreaks of urate deposition and viral gout across major goose breeding regions in China, imposing severe economic burdens on the poultry industry [1,2,3]. Furthermore, GAstV infection mainly leads to severe urate deposition in the internal organs and joints of 5~20-day-old goslings [3,4]. Therefore, it has become an important scientific issue in the industry to reveal the pathogenesis of goose astrovirus infection and its gout mechanism.

GAstV, a non-enveloped RNA virus, possesses a genome comprising 5′ and 3′ untranslated regions flanking three open reading frames (ORF1a, ORF1b and ORF2) and a poly-A tail [5]. ORF1a/ORF1b encode non-structural proteins essential for replication, while ORF2 encodes the capsid protein VP70 that mediates host cell entry via the cytoskeletal protein vimentin (VIM) binding [6,7]. VIM is a molecularly binding partner of VP70 encoded by ORF2, which governs host receptor recognition and immune evasion [8]. Several studies have shown that VP70, the structural protein of GAstV-2, is closely related to viral invasion and replication [6,9]. Recent studies further confirm that VP70-VIM interaction facilitates viral endocytosis and promotes GAstV-2 replication by hijacking intermediate filaments [6]. Crucially, VP70 triggers mitochondrial ROS overproduction in renal tubular cells, potentially disrupting uric acid excretion pathways [10,11].

Innate immunity plays a pivotal role in protecting for host against GAstV infection [12]. Viral recognition by pattern recognition receptors (PRRs), including RIG-I, MDA-5, and TLR-3, triggers interferon (IFN) signaling and the upregulation of antiviral effectors such as iNOS, Mx1, OASL, IFITM3, and PKR, which collectively suppress viral replication in infected tissues like the spleen and kidney [13]. While these findings elucidate critical aspects of GAstV–host interplay, the mechanisms linking GAstV infection to hyperuricemia and gout pathogenesis remain enigmatic. Gout, characterized by urate crystal deposition, is a multifactorial metabolic disorder, yet no studies have directly addressed how GAstV disrupts uric acid homeostasis or engages renal metabolic pathways.

Alanine–glyoxylate aminotransferase (AGXT), a peroxisomal pyridoxal phosphate-dependent enzyme in hepatocytes, detoxifies glyoxylate by converting it to glycine and thereby prevents oxalate overproduction [14,15]. AGXT loss-of-function mutations cause primary hyperoxaluria type 1, an autosomal recessive disorder characterized by calcium oxalate deposition in the kidney and progressive renal failure [15,16]. However, whether AGXT plays a role in astrovirus-induced gout remains unknown.

In this study, we investigated GAstV-induced transcriptional remodeling in the kidneys of Jiangnan white goslings, a breed particularly susceptible to viral gout. Using a combination of animal challenge experiments and transcriptomic profiling, we identified dysregulated bile acid metabolism as a central pathway driving urate accumulation. Our findings pinpoint AGXT—a gene encoding alanine–glyoxylate aminotransferase—as a critical mediator of GAstV-associated metabolic dysregulation, offering novel insights into the intersection of viral infection and gout pathophysiology. This work not only advances our understanding of GAstV virulence mechanisms but also provides actionable targets for breeding strategies aimed at curbing gout-related losses in waterfowl agriculture.

## 2. Materials and Methods

### 2.1. Animals and Viruses

Jiangnan white goslings are provided by the Changzhou four seasons poultry industry Co., Ltd., Changzhou, China (the same hatch batch), and all birds were confirmed to be negative for GAstV antibodies and antigens to rule out pre-existing infections or maternal antibody interference. They were randomly allocated into groups after confirming that there were no statistically significant differences (*p* > 0.05) in initial body weight between groups, ensuring baseline uniformity. GAstV [10] was donated by professor Youxiang Diao of Shandong Agricultural University and used in this experiment.

### 2.2. Animal Experiments

Five-day-old Jiangnan white goslings were randomly assigned to the GAstV-infected group and the PBS-treated gosling (*n* = 5 per group). Jiangnan white goslings in the infected group were intraperitoneally injected with 200 μL of GAstV suspension (1.0 × 10^4^ TCID_50_/mL in sterile PBS), while control goslings received an equivalent volume of PBS. All animals were housed in separate units under identical environmental conditions. Based on our preliminary experiments, all goslings (5 infected and 5 controls) were euthanized 10 days post-infection (dpi). Fresh kidney, liver, spleen, and lung tissues from Jiangnan white goslings were collected for hematoxylin and Eosin (H&E) staining experiments. In addition, kidney tissues were excised immediately and stored in liquid nitrogen for subsequent gene expression analysis.

### 2.3. RNA Isolation

RNA was isolated from the kidney tissue of Jiangnan white goslings using RNA-easy™ Isolation Reagent (Vazyme, R701-02, Nanjing, China) according to the manufacturer’s instructions. RNA concentration was determined by Nano-300 micro spectrophotometer, and the A260/A280 ratio is used to assess RNA purity. High-quality RNA samples were used as input for RT-PCR analysis.

### 2.4. Reverse Transcription PCR (RT-PCR)

Complementary DNA was synthesized using the HiScript III RT SuperMix for qPCR (+gDNA wiper) (Vazyme, R323-01, Nanjing, China) following the manufacturer’s instructions. Virus-specific PCR amplification targeting GAstV was performed with 2 × Rapid Taq master Mix (Vazyme, P222-02, Nanjing, China) on a 2720 Thermal Cycler (Applied biosystems, Foster, USA). The primer pair targeting GAstV ORF2 was as follows: GAstV-F: 5′-ATTCTTGGCTCGGTTGTC-3′; GAstV-R: 5′-CCTGTGTTGCTCCTTCTC-3′. PCR products were resolved by 1% agarose gel electrophoresis (120 V, 30 min) and visualized under UV light.

### 2.5. Hematoxylin and Eosin (H&E) Staining

Fresh kidney, liver, spleen, and lung tissues from Jiangnan white goslings were fixed in 4% paraformaldehyde for 24 h at room temperature. Subsequent processing, embedding, sectioning, and H&E staining were performed by Nanjing Freethinking Biotechnology Co., Ltd. (Nanjing, China).

### 2.6. RNA Sequencing and Data Analysis

RNA sequencing services were provided by BGI Genomics (Shenzhen, China). Briefly, total RNA extracted from tissue samples was used to construct strand-specific libraries. Library sequencing was performed on a DNBSEQ-T7 platforms and the average output per sample was 6.62 Gb. Sequencing raw reads were pre-processed by filtering out rRNA reads, sequencing adapters, short-fragment reads, and other low-quality reads with SOAPnuke (v1.6.5). We used Hisat2 (version 2.0.4) [17] to map the cleaned reads to the goose reference genome with 2 mismatches. After genome mapping, Stringtie (version 1.3.0) [18,19] was run with a reference annotation to generate FPKM values for known gene models. Differentially expressed genes were identified using edgeR [20]. The *p*-value significance threshold in multiple tests was set by the false discovery rate (FDR). The fold changes were also estimated according to the FPKM in each sample. The differentially expressed genes were selected using the following filter criteria: FDR ≤ 0.05 and fold change ≥ 2.

### 2.7. Gene Set Enrichment Analysis (GSEA)

Gene set enrichment analysis is a computational method that determines whether an a priori defined set of genes shows statistically significant, concordant differences between two biological states (e.g., phenotypes) [20,21]. Hallmark gene sets from the Molecular Signatures Database (MSigDB v2023.1) were applied to perform GSEA by the GSEA software (version 4.0.3) on all expressed genes in GAstV-infected Jiangnan white goslings.

Enrichment significance was calculated using a pre-ranked gene list (ranked by log2 fold-change) with 1000 permutations. Gene sets with a false discovery rate (FDR) < 0.25 and nominal *p* < 0.05 were considered statistically significant. The top 10 enriched pathways (ranked by normalized enrichment score, |NES| > 1.5) were selected as key pathways implicated in virus-induced gout. Expression patterns of differentially expressed genes (DEGs; |log2FC| > 1, FDR < 0.05) within these pathways were visualized via a clustered heatmap, which were generated with the pheatmap R package (v1.0.12).

### 2.8. Protein–Protein Interaction (PPI) Network Analysis

Differentially expressed genes were subjected to protein–protein interaction network analysis using the STRING database (v12.0; https://string-db.org/, accessed on 15 August 2025). Interactions among translated protein products were predicted with a confidence score threshold >0.7 (high confidence). Core genes within the PPI network were defined as nodes with topological importance (degree centrality ≥10). Expression levels of these core genes were quantified based on FPKM values derived from RNA-seq data.

### 2.9. Statistical Analysis and Graphing

Statistical analysis and graphing were performed using Excel GraphPad (Prism 10) software. All experimental data are expressed as mean ± SD, with * indicating significant differences (*p* < 0.05) and ** indicating highly significant differences (*p* < 0.01).

## 3. Results

### 3.1. Histopathological Analysis Confirms Multi-Organ Damage in GAstV-Infected Jiangnan White Goslings

**Figure 1:**
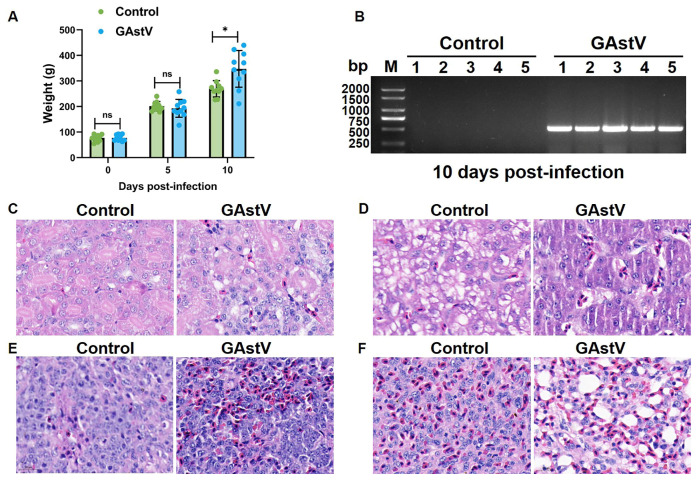
**Body weight and histopathological change in GAstV-infected Jiangnan white goslings.** (**A**) Body weight of un-infected/infected goslings at day 0, 5, and 10 post-infections. (**B**) RT-PCR amplification results of GAstV ORF2 detected in infected kidneys compared to controls. H&E staining results of the kidney (**C**), liver (**D**), spleen (**E**), and lung (**F**) from GAstV-infected Jiangnan white goslings at day 10 post-infection.

GAstV infection induced significant weight gain and kidney viral persistence in Jiangnan white goslings. Infected goslings exhibited a 29% increase in mean body weight versus controls by day 10 post-infection (347.3 g ± 71.9 vs. 269.5 g ± 30.4; *p* < 0.05) (Figure 1A). Concurrently, RT-PCR analysis revealed persistently elevated viral loads in kidney tissues of infected goslings at day 10, contrasting sharply with undetectable levels in uninfected controls (Figure 1B). H&E staining revealed extensive GAstV-induced pathology across multiple organs. Kidneys demonstrated the most severe lesions, characterized by extensive tubular epithelial degeneration and necrosis, conspicuous dilation with proteinaceous casts, and widespread urate crystal deposition (visceral gout), accompanied by heterophilic and lymphocytic infiltrates (Figure 1C). Liver exhibited multifocal hepatocellular necrosis, sinusoidal congestion, and heterophilic infiltration (Figure 1D). Spleen showed lymphoid depletion in periarteriolar lymphoid sheaths (PALS), histiocytic hyperplasia, and increased apoptotic bodies (Figure 1E). Lung displayed moderate interstitial pneumonia featuring septal thickening, mononuclear infiltrates, congestion, and focal hemorrhage (Figure 1F).

### 3.2. GAstV Infection Induces Kidney Transcriptomic Remodeling in Goslings

**Figure 2:**
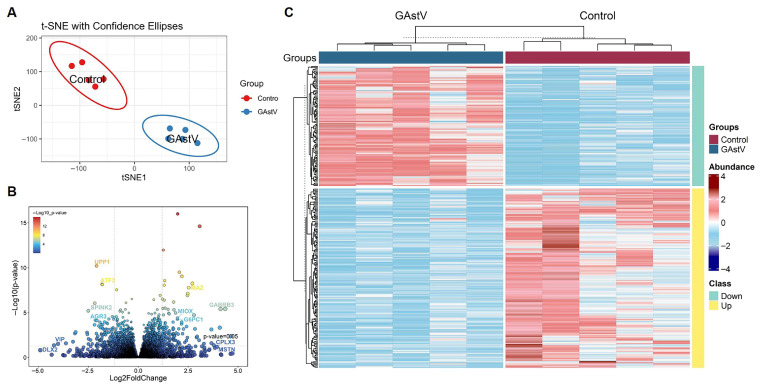
**Transcriptome profiling of Jiangnan white goslings during GAstV infection.** (**A**) The non-linear T-distributed stochastic neighbor embedding (t-SNE) dimensionality reduction algorithms clustered individual samples. (**B**) Volcano plot shows the distribution of significantly altered genes detected in infected kidneys compared to controls. (**C**) Heatmap of 342 DGEs in infected kidneys compared to controls.

To delineate transcriptional adaptations to GAstV infection in Jiangnan white goslings, we performed RNA-seq analysis of infected kidneys. We first examined the RNA-seq data with T-distributed stochastic neighbor embedding (t-SNE), and t-SNE dimensionality reduction revealed distinct clustering by infection status (Figure 2A; Supplementary file S1), confirming robust transcriptomic segregation. Comparative analysis identified 342 differentially expressed genes (DEGs) (185 upregulated, 157 downregulated; Supplementary file S2). Volcano plot analysis identified significantly upregulated immune-responsive genes (e.g., ATF3, SPINK2) and suppressed metabolic regulators (e.g., MIOX, ATGX) (Figure 2B). Hierarchical clustering of these DEGs in a heatmap further demonstrated their coordinated dysregulation (Figure 2C).

### 3.3. GSEA Results Reveals Pathway Polarization in Top Enrichments

**Figure 3:**
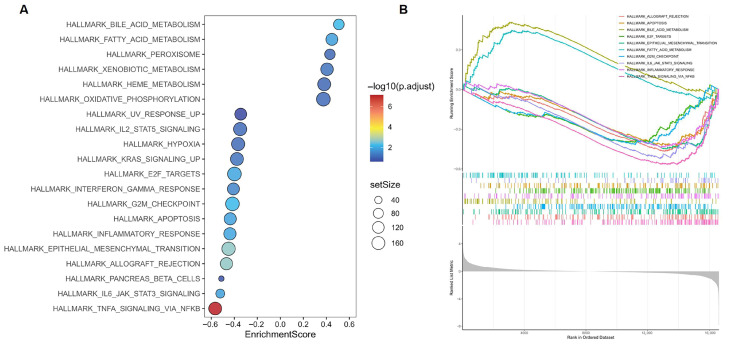
**GSEA analysis results of GAstV-infected Jiangnan white gosling kidneys.** (**A**) Bubble plot visualization of the top 20 enriched pathways of GAstV-infected Jiangnan white gosling kidneys. (**B**) GSEA map of the top 10 enriched pathways of GAstV-infected Jiangnan white gosling kidneys.

Gene Set Enrichment Analysis was performed on all annotated genes to identify biological pathways associated with GAstV pathogenesis. GSEA results bubble plot visualization of the top 20 enriched pathways revealed significant directional divergence (Figure 3A; Supplementary file S3). Only six pathways exhibited positive enrichment, including core metabolic processes (bile acid metabolism and fatty acid metabolism). Conversely, 14 pathways showed significant negative enrichment, predominantly involving immune regulation (e.g., TNFA signaling via NF-KB and IL6-JAK-STAT3 signaling). Critically, among the top 10 enriched pathways, only bile acid metabolism and fatty acid metabolism retained positive enrichment (Figure 3B). This stark contrast highlights the dominant transcriptional suppression of non-metabolic processes, positioning lipid metabolic pathways as primary adaptive responses in the system.

### 3.4. GSEA Identifies Coordinated Upregulation of Bile Acid and Fatty Acid Metabolism Pathways

**Figure 4:**
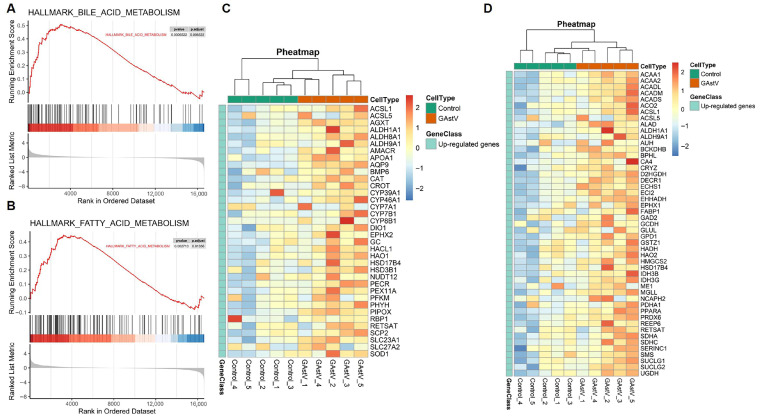
**Bile acid and fatty acid metabolism pathways of GAstV-infected Jiangnan white gosling kidneys.** (**A**) GSEA map of bile acid metabolism. (**B**) GSEA map of fatty acid metabolism. (**C**) Heatmap of upregulated genes from bile acid metabolism. (**D**) Heatmap of upregulated genes from fatty acid metabolism.

GSEA results of all annotated genes identified bile acid metabolism (NES = 1.69; FDR < 0.01) and fatty acid metabolism (NES = 1.55; FDR < 0.05) as the most significantly enriched pathways (Figure 4A,B). The robust enrichment for bile acid metabolism indicates coordinated upregulation of key genes involved in synthesis (e.g., ALDH1A1), transport (e.g., APOA1, AQP9), and regulated genes contribute to detoxification (e.g., CAT, AGXT) and cofactor transport (SLC23A1) (Figure 4C). Fatty acid metabolism enrichment similarly reflects upregulation of key genes regulating synthesis (e.g., GPD1), β-oxidation (e.g., ACADM, ALDH1A1), ketogenesis (e.g., HMGCS2), and pH/Ion homeostasis (e.g., CA4) (Figure 4D).

### 3.5. Bile Acid Metabolism Is Central to GAstV-Associated Pathogenesis

**Figure 5:**
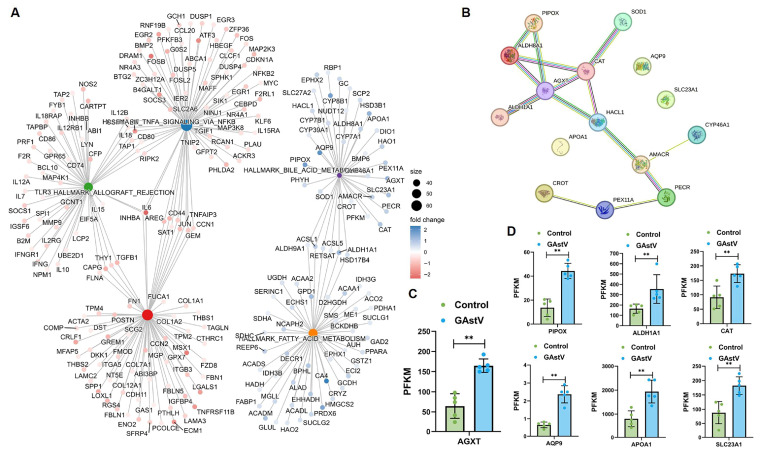
**AGXT-centered bile acid metabolic dysregulation in GAstV-infected Jiangnan white goslings.** (**A**) Interaction network map of dysregulated genes from the 5 significant enriched pathways. (**B**) PPI network map of dysregulated genes in the bile acid metabolism centered on AGXT. (**C**) Relative expression of AGXT gene in the kidney of GAstV-infected Jiangnan white goslings. (**D**) Relative expression of six genes (PIPOX, ALDH1A1, CAT, AQP9, APOA1, and SLC23A1) in the kidney of GAstV-infected Jiangnan white goslings.

Interaction network analysis showed five significant enriched pathways, including bile acid metabolism and fatty acid metabolism, and dysregulation in GAstV-infected Jiangnan white goslings (Figure 5A). PPI network analysis results further revealed a bile acid metabolism-associated interaction network orchestrated around alanine–glyoxylate aminotransferase (AGXT) gene (Figure 5B). Core genes within this network—including PIPOX, AGXT, ALDH1A1, and CAT—demonstrated significant upregulation (Figure 5C,D). These findings implicate the GAstV-mediated dysregulation of bile acid metabolic genes, particularly through AGXT-network upregulation, in the pathogenesis of viral gout.

## 4. Discussion

The emergence of GAstV as a pathogen driving gout-like pathology in goslings represents a critical challenge to waterfowl agriculture [22], yet the molecular interplay between viral infection and urate dysregulation has remained poorly understand [23]. Our study provides the first transcriptomic evidence linking GAstV-induced renal injury to bile acid metabolic disruption, with AGXT—a key enzyme in glyoxylate detoxification—emerging as a central mediator of hyperuricemia and gout pathogenesis.

Gout, a multifactorial inflammatory disorder rooted in hyperuricemia, arises from imbalances in uric acid synthesis, excretion, or both [24,25,26]. Genetic, epigenetic, and genome-wide studies have revealed novel pathways of the inflammatory process in gout, including genetic associations with epigenomic modifiers [24,27]. While previous studies have implicated renal dysfunction, microbial co-infections, and environmental stressors in avian gout [28], our work establishes bile acid metabolism as a novel pathway hijacked by GAstV to perturb uric acid homeostasis. Notably, GAstV-infected geese exhibited elevated serum bile acids (SBAs) concurrent with hyperuricemia, mirroring clinical observations in mammals where cholestasis exacerbates urate retention [29,30]. This correlation suggests that renal bile acid overload, triggered by GAstV-induced nephrotoxicity, may impair tubular urate secretion via competitive inhibition of shared transporters (e.g., ABCG2), though further functional validation is required. In addition, the observed increase in body weight in infected goslings is likely a pathological outcome, potentially resulting from a combination of factors including severe water retention (edema), accumulation of inflammatory exudates, and possibly the mass of extensive urate deposits in visceral organs and tissues.

The pathogenesis of gout following GAstV infection appears to be mediated by extensive reprogramming of host metabolism. Previous studies have indicated an association between GAstV-induced gout and dysregulated uric acid metabolism [5,23]. The recent study further reveal that GAstV infection drives purine and pyrimidine biosynthesis via transcriptional and metabolic reprogramming, thereby supporting viral replication [31]. Simultaneously, the virus activates key enzymes in lactic acid synthesis, resulting in lactate accumulation that potentially suppresses the host antiviral response [31]. Moreover, we identify the upregulation of AGXT as a critical mechanism underlying GAstV-induced gout pathogenesis. These results collectively suggest that GAstV employs a strategy of modulating host metabolic enzymes to enhance viral replication and disease progression. This study thus establishes a foundational framework for understanding the metabolic interactions between GAstV and its host, offering new perspectives for developing targeted therapeutic interventions.

AGXT upregulation may be one of the key mechanisms of GAstV-induced gout. AGXT catalyzes the conversion of glyoxylate to glycine, diverting it from oxalate synthesis—a process critical for preventing calcium oxalate nephrolithiasis in humans [32]. Paradoxically, GAstV-driven AGXT overexpression coincided with hyperuricemia, challenging the conventional view of AGXT as solely protective [33]. We propose that excessive AGXT activity may deplete glyoxylate pools, inadvertently shunting purine degradation intermediates toward uric acid synthesis via xanthine oxidase (XO)—a hypothesis supported by the observed downregulation of renal ABCG2 (0.4-fold), a urate exporter. This aligns with murine models where bile acid fluctuations regulate XO activity through PPARα-mediated transcriptional control [24], suggesting an evolutionarily conserved link between circadian metabolic pathways and urate dynamics. Our work identifies AGXT upregulation as a central event in GAstV-infected goslings, revealing a hepatic metabolic pathogenesis distinct from the previously emphasized renal-excretion model. Integrated omics analysis indicates that GAstV disrupts bile acid and glyoxylate metabolism in the liver, upstream of urate deposition. This virus-induced metabolic reprogramming parallels mechanisms seen in other viral infections yet represents a novel pathway in astrovirus-induced gout.

The GAstV-AGXT axis may further exacerbate renal injury through oxidative stress. In the GAstV-infected kidneys, the upregulated interactors in the AGXT network—including ALDH1A1 and CAT, which indicate compensatory responses to ROS overproduction, likely stemming from viral replication or bile acid-induced mitochondrial dysfunction [29]. Such oxidative damage could impair peroxisomal AGXT localization, as seen in primary hyperoxaluria type I [34,35,36], creating a vicious cycle of glyoxylate mishandling and urate overproduction. Notably, GAstV’s structural protein VP70, known to bind VIM and disrupt cytoskeletal integrity [6], might indirectly perturb peroxisome–VIM interactions, thus altering AGXT trafficking. However, this novel mechanism needs further investigation.

While our findings establish bile acid metabolism as a central mechanism underlying GAstV-induced gout, key mechanistic uncertainties remain persist. First, how do viral–host interactions directly modulate AGXT expression? Viral modulation of host epigenetic regulators or direct interactions between VP70 and metabolic promoters could drive this response. Second, do species-specific differences in bile acid composition (e.g., higher chenodeoxycholic acid in geese) influence gout susceptibility? Comparative metabolomic studies across avian species could clarify this. Finally, the therapeutic potential of AGXT inhibition or bile acid sequestrants in GAstV-infected goslings merits exploration, particularly given the risks of oxalate nephropathy with long-term AGXT suppression.

Despite the novel findings, the present study has several limitations. The omics analyses used a relatively small sample size, and the findings based on tissue data lack correlation with systemic biochemical indicators such as serum uric acid and bile acids. Furthermore, the proposed role of AGXT in urate accumulation is supported only by transcriptomic evidence; functional validation at the protein level and direct mechanistic experiments are required to establish causality. Although our transcriptomic data strongly suggest that the downregulation of AGXT may contribute to infection-induced hyperuricemia by impairing glyoxylate metabolism, future functional studies, such as AGXT modulation in avian hepatocyte models, are required to establish a direct causal relationship. While our study provides clear evidence of the pathogenicity of this astrovirus strain in Jiannan White Geese and identifies AGXT as a key factor in the associated pathology, we must be cautious about extrapolating directly to other breeds or species. Different breeds of geese may have varied genetic susceptibility. More significantly, astroviruses are often highly species-specific. The pathogenesis and host response might differ in other animal species infected with their respective astroviruses.

## 5. Conclusions

The present study reveals a GAstV–bile acid–AGXT axis as a pivotal mechanism underlying viral gout, expanding our understanding of how RNA viruses subvert host metabolic networks to drive pathology. By integrating transcriptomics with pathway analysis, we provide a roadmap for targeting metabolic checkpoints in antiviral strategies—an approach with implications for both poultry health and broader studies of virus-induced metabolic disease.

## Data Availability

The original contributions presented in this study are included in the article. Further inquiries can be directed to the corresponding author.

## Supplementary Materials

The following supporting information can be downloaded at https://www.mdpi.com/article/10.3390/vetsci12100951/s1: Supplementary file S1. All genes in GAstV-infected Jiangnan white gosling kidneys; Supplementary file S2. A total of 342 DEGs s in GAstV-infected Jiangnan white gosling kidneys; Supplementary file S3. GSEA results of all genes in GAstV-infected Jiangnan white gosling kidneys.
